# Usage of Net Promoter Score in Medical Education Literature: A Scoping Review

**DOI:** 10.7759/cureus.95919

**Published:** 2025-11-01

**Authors:** Yuya Takahashi, Nobukazu Agatsuma, Fumiya Oguro, Mitsuaki Oura, Fareeda Abo-Rass, Yoshitaka Nishikawa

**Affiliations:** 1 Integrated Clinical Education Center, Kyoto University Hospital, Kyoto, JPN; 2 Department of Gastroenterology and Hepatology, Kyoto University Graduate School of Medicine, Kyoto, JPN; 3 Department of Internal Medicine, Hirata Central Hospital, Fukushima, JPN; 4 Division of Hematology/Oncology, Kameda Medical Center, Chiba, JPN; 5 Department of Social Work, Ben-Gurion University of the Negev, Be'er Sheva, ISR; 6 Takemi Program in International Health, Harvard T.H. Chan School of Public Health, Boston, USA; 7 Department of Health Informatics, Kyoto University School of Public Health, Kyoto, JPN

**Keywords:** evaluation metrics, lifelong learning, medical education, net promoter score, scoping review

## Abstract

Net Promoter Score (NPS^®^) has been widely used in industry to measure customer loyalty, but its use in medical education has not been systematically explored. This scoping review examined how NPS has been used to evaluate educational interventions in medical education. We searched MEDLINE/PubMed and Scopus for studies involving medical students and doctors as participants without restriction on the publication year and identified 19 eligible articles. The review clarified a wide range of applications of NPS, often in combination with other assessment tools, such as Likert scales or open-ended feedback, but the number of reviewed studies that explored the reasons behind NPS was limited. In addition, 12 out of 19 studies employed NPS to measure general satisfaction rather than its original purpose of gauging loyalty or recommendation. Furthermore, 13 studies followed the original calculation method, highlighting both a lack of awareness of the original purpose of NPS and inconsistencies in its application. Although focusing on English-language papers may provide a biased overview, this review suggests opportunities for the consistent use of NPS and focuses on NPS reasons and its correlation with other metrics. Adopting these approaches will facilitate future comparisons of educational interventions and enable the exploration of meaningful insights into enhancing educational quality.

## Introduction and background

Medical education is a dynamic and continuous process that begins with basic medical education (what medical students learn in medical school), continues through residency and specialty training, and extends throughout their careers, ultimately leading to lifelong learning [1]. The primary objective of medical education is to equip physicians with the skills and knowledge necessary to provide high-quality care, promote health, prevent disease, and alleviate symptoms [1]. The quality of medical education is increasingly required to improve, and it directly affects the quality of medical care physicians can provide, as a past report has shown that those who receive superior education are more likely to achieve better patient outcomes [2]. Therefore, to enable medical students and doctors to provide high-quality care, enhancing the quality of medical education is essential.

One effective way to improve the quality of education is to evaluate educational methods and processes regularly [3]. Medical education increasingly requires evaluation as part of its quality assurance procedures, and various assessment techniques have been employed to evaluate medical education programs and training, such as interviews (two-person meeting style), student ratings, open-ended questions (free-form writing via questionnaire), and scaled questionnaires [4,5]. However, interviews with large groups are often impractical because of the time and effort required, and student ratings can be highly dependent on individual abilities and motivation. As a result, questionnaire surveys have become the most commonly used assessment tool using objective measurements [5].

Net Promoter Score (NPS^®^), one of the scalable metrics, has been widely used since 2003 in various fields, such as software evaluation and customer experience research, and is frequently employed to measure customer loyalty or predict growth [6-8]. The NPS question “How likely are you to recommend X to a friend (or colleague)?” is rated from 0 to 10, and the responses are categorized into detractors (≤ 6), passives (7,8), and promoters (9,10). NPS is calculated by subtracting the percentage of detractors from the percentage of promoters [7,8].

Although NPS has been shown to be useful in other fields (e.g., customer loyalty), there have been few systematic reviews of its use in medical education. Previous research has focused almost exclusively on continuing medical education (CME) for medical professionals (mainly physicians) [9], with little attention to its use among medical students - a critical yet underexplored group within the continuum of medical education. NPS is a standard scale with a simple question, and it has the drawback of not providing information for improvement. It has unique characteristics that other measures do not, such as enabling straightforward analysis and serving as a benchmark for evaluating the value of interventions [7]. Given these characteristics, it may be worthwhile to examine whether NPS could serve as an effective indicator because it has the potential to be an attractive indicator in medical education when assessing the value of educational interventions. Investigating the current usage of NPS in medical education and understanding its usefulness, advantages, and limitations will clarify what is important and what needs to be considered for future applications. Therefore, this scoping review aims to explore how NPS has been applied, interpreted, and assessed in educational interventions among medical students and doctors. In addition, it seeks to synthesize the existing literature to provide insights into the practical use of NPS as an assessment tool in medical education and to explore additional assessment methods used alongside NPS.

## Review

Methods

This review followed the Preferred Reporting Items for Systematic Reviews and Meta-Analyses for Scoping Reviews (PRISMA-ScR) guidelines [10].

Search Strategy

We sourced articles from MEDLINE/PubMed and Scopus databases. The search was conducted on August 23, 2025. The first author (YT) and co-author (YN) developed a search strategy encompassing synonymous terms related to NPS and education using standard Medical Subject Headings (MeSH) terms and thesaurus (see Table 1). The identified studies were screened according to the PRISMA 2020 flow diagram. Since NPS was introduced in 2003 [8] and available documents were expected to be limited, we did not set any restrictions on the publication year to ensure comprehensive research. We also excluded articles unavailable in English and certain types of articles, such as conference papers.

**Table 1:** Search formula for the identification of studies

Database	Search formula
PubMed	(“education"[MeSH Terms] OR "teach*"[Title/Abstract] OR "train*"[Title/Abstract] OR "program*"[Title/Abstract] OR "develop*"[Title/Abstract] OR "educat*"[Title/Abstract]) AND "net promoter*"[Title/Abstract]
Scopus	(TITLE-ABS-KEY ("net promoter*") AND TITLE-ABS-KEY ("educat*" OR "train*" OR "teach*" OR "develop*" OR "program*"))

Study Selection

Articles were included if they explicitly mentioned the use of NPS and were relevant to the medical field. Studies were excluded if they did not focus on education, lacked sufficient involvement of medical students or doctors (e.g., studies in which the main participants were other medical professionals such as nurses, veterinarians, and dentists), or used NPS to evaluate medical therapies or technologies instead of educational interventions. Throughout the screening process, we applied both inclusion and exclusion criteria. The first author (YT) and co-author (YN or NA) independently conducted primary screening of titles and abstracts, and the studies that only one of the two researchers judged as eligible were subject to the second screening unless our discussion determined them to be clearly ineligible. The first (YT) and co-authors (NA, FO, MO, and YN) also independently conducted secondary full-text screening and made a final decision by discussing together when there was a disagreement about whether to include a study in the review. We compiled the identified articles from MEDLINE/PubMed and Scopus in Excel and removed duplicates with DOI and article titles. We also calculated Cohen’s κ coefficient as an indicator of inter-rater reliability using EZR^®^.

Data Extraction and Analysis

The first (YT) and co-author (YN) jointly developed a data-charting form and determined the variables to be extracted. The first (YT) and co-authors (NA, FO, MO, and YN) independently extracted data from the studies, discussed the results, and iteratively refined the data-charting form. When discrepancies arose in the data extracted by each author, discussions were held to reach a consensus on interpretation. We extracted data on several key elements, including the first author, journal, year of publication, country or region, the subject evaluated using NPS, study setting, domain of educational intervention, participant characteristics, a description of the intervention, study design, timing of assessment, the purpose of NPS usage, and any associated evaluation measures. In addition, we focused on the question format, response method, and calculation method for NPS, as well as whether there were any descriptions related to loyalty and satisfaction in connection with NPS. Finally, we did not conduct a formal risk of bias assessment, as it is not required for scoping reviews according to PRISMA-ScR and the JBI Manual for Evidence Synthesis [11,12].

Results

**Figure 1:**
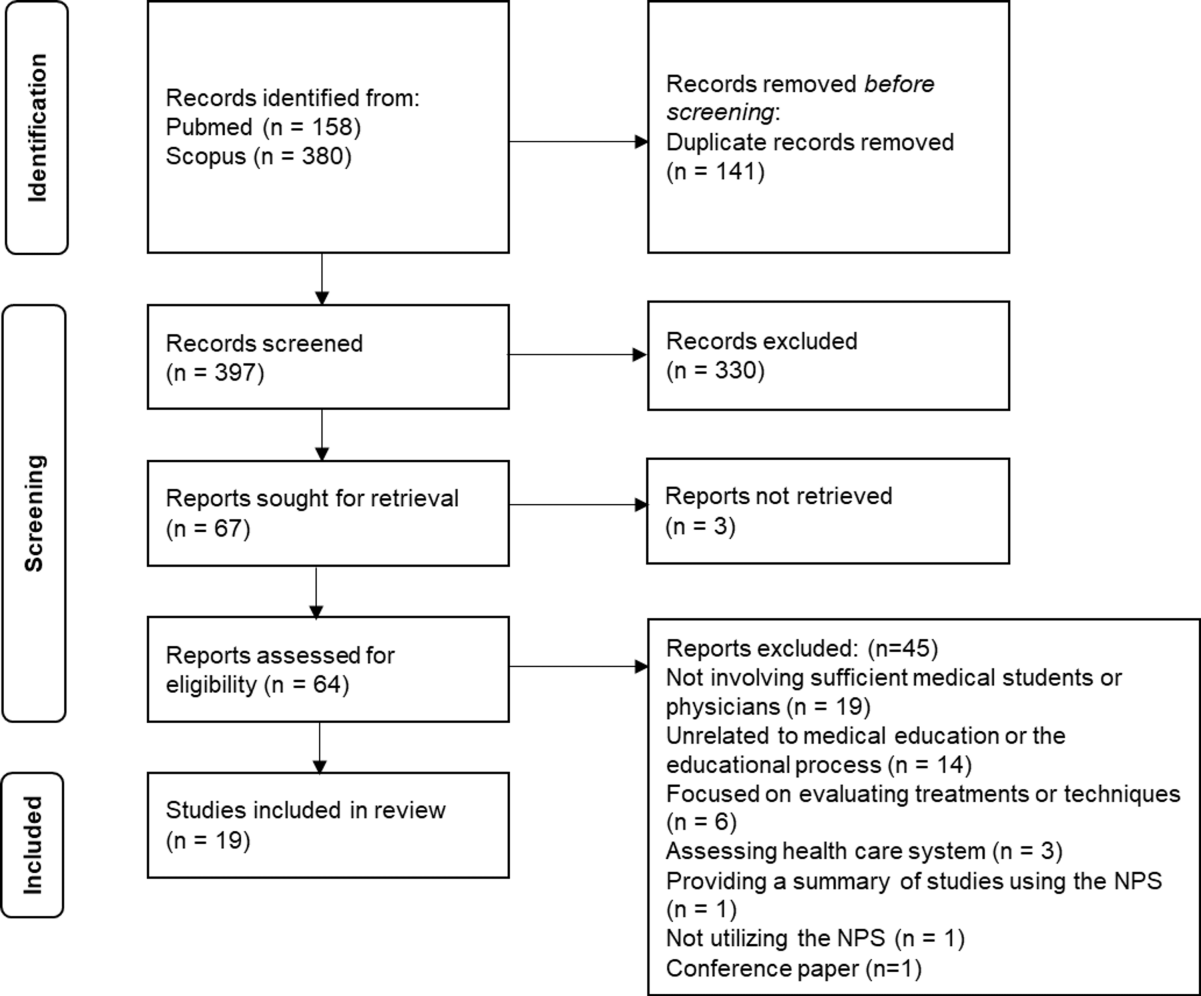
PRISMA flow diagram for the study selection PRISMA: Preferred Reporting Items for Systematic Reviews and Meta-Analyses

A total of 538 records were retrieved from the database searches, and 19 full-text articles were included in the review following the PRISMA-ScR guideline (see Figure 1). Details of the screening process and reasons for exclusion are provided in the Appendices. Cohen’s κ coefficient was 0.69 for the primary screening and 0.36 for the secondary screening, and it reached 1.0 after discrepancies were resolved through discussion.

Characteristics of the Studies

Among the 19 selected studies (see Table 2) [13-31], 15 (79%) were conducted in the United States, with the remaining four in France [24], Canada [25], Switzerland [29], and Brazil [30]. The publication year of the reviewed study ranged from 2018 to 2025.

**Table 2:** Characteristics of the selected studies Workplace-based learning is training in clinical settings that does not focus on the development of skills directly related to patient care.

Reference	Journal	Year of publication	Country	Setting	Domain of educational intervention	Participants	Intervention	Study design	Assessment timing	Purpose of NPS usage	Associated measures
Sun et al. [13]	Plast Reconstr Surg Glob Open.	2018	USA	Coaching	Plastic surgery	406 American Council of Academic Plastic Surgeons faculty members	Plastic surgery residency program	Surveys about the existing educational system	Post-intervention	To categorize responses to valuation questions	Likert scale question
Sieja et al. [14]	Mayo Clin Proc.	2019	USA	Workplace-based learning	Electronic health record	220 clinicians	The Sprint intervention to learn EHR	Intervention for one group	Pre- and post-intervention	To assess participants' satisfaction	The emotional exhaustion domain from the Maslach Burnout Inventory, Likert scale questions
Smithson et al. [15]	J Med Educ Curric Dev.	2020	USA	Basic medical education	Basic medical education	18 fourth-year medical students	A simulation and two sessions to develop communication, leadership, and teamwork skills	Intervention for one group	Post-intervention	To gauge the level of overall satisfaction	Likert scale questions
Klifto et al. [16]	Plast Reconstr Surg Glob Open.	2021	USA	Coaching	Plastic surgery	124 in the in-person course and 770 in the virtual course, including students and researchers	Pennsylvania Flapping Course focusing on education and techniques of dissections pertinent to flap-based reconstruction	Intervention for two groups	Post-intervention	To assess participants' satisfaction & for course satisfaction and loyalty to the virtual interface	NA
Lourie et al. [17]	J Am Med Inform Assoc.	2021	USA	Workplace-based learning	Electronic health record	451 participants, including physicians, physician fellows	The program about EHR	Intervention for one group	Post-intervention	To assess how likely participants are to recommend the program	Likert scale questions
Pradarelli et al. [18]	JAMA Surg.	2021	USA	Coaching	Surgery	46 practicing surgeons	SCOPE program to learn surgical technique	Intervention for one group	Post-intervention	To indicate how likely they were to recommend their coaching session to others	NA (interview)
Sanseau et al. [19]	AEM Educ Train.	2021	USA	Basic medical education	Emergency medicine	67 medical students and 29 facilitators (including 28 physicians)	Twenty-one telesimulations related to emergency medicine	Intervention for one group	Post-intervention	To measure user satisfaction overarchingly	SET-M, Likert scale questions
Moschovis et al. [20]	BMC Med Educ.	2022	USA	Basic medical education	Global health	32 students and 18 guests	Ten online sessions to learn global health and clinical skills	Intervention for one group	Post-intervention	To determine participants' likelihood of recommending the course	Likert scale questions, Open-ended questions
Kaur et al. [21]	AEM Educ Train.	2023	USA	Coaching	Emergency medicine	75 participants (including 41 nurses and 17 physicians)	Twenty-four three-hour simulation sessions about pediatric emergencies	Intervention for one group	Post-intervention	Not described in the manuscript	Likert scale questions, Multiple-choice questions, Multiple-option-answer questions
Sharpe et al. [22]	J Am Coll Radiol.	2023	USA	Coaching	Radiology	1153 ACR members (including 1036 practicing physicians, 66 residents or fellows)	Peer learning that aims to surface learning opportunities	Surveys about the existing educational system	Post-intervention	To determine user satisfaction	Multiple-option-answer questions
Yee et al. [23]	Pediatr Emerg Care.	2024	USA	Coaching	Pediatric emergency medicine	118 participants (including 100 physicians, 7 fellows, four residents)	E-book about pediatric emergency medicine	Intervention for one group	Post-intervention	Not described in the manuscript	Likert scale questions, Multiple-choice questions, Open-ended questions
Descamps et al. [24]	Hand Surg Rehabil.	2024	France	Coaching	Surgery	94 doctors (90 orthopedic surgeons and 4 plastic surgeons)	Three key seminars about upper-limb ultrasound	Intervention for one group	Post-intervention	To measure social acceptance amongst learners and facilitators of the VRR platform	SET-M
Leung et al. [25]	AEM Educ Train.	2024	Canada	Coaching	Emergency medicine	90 emergency medicine residents	Two days of a total of six cases from EM ReSCu Peds	Intervention for one group	Post-intervention	To assess provider satisfaction	Time spent and financial data
Chen et al. [26]	Appl Clin Inform.	2024	USA	Workplace-based learning	Electronic health record	32 primary care physicians, 28 specialty physicians, and 16 nonphysician providers	Seventy-six sessions about EHR	Intervention for one group	Post-intervention	To assess simulation satisfaction	Likert scale questions
Athanasopoulou et al. [27]	Cureus.	2024	USA	Coaching	Emergency medicine	33 facilitators (including 26 pediatric emergency physicians) and 55 learners (including 46 paramedics)	A unique SimBox case about the resuscitation of a newborn baby	Intervention for one group	Post-intervention	To determine participants' likelihood of recommending the course	Likert scale questions, Open-ended questions
Shin et al. [28]	J Educ Teach Emerg Med.	2025	USA	Basic medical education	Emergency medicine	78 learners included medical students, junior residents, senior residents and so on	Seven two-hour emergency simulations with backpack	Intervention for one group	Post-intervention	To assess satisfaction and learning experiences	NA(Free comments)
Saad et al. [29]	Klin Monbl Augenheilkd.	2025	Switzerland	Coaching	Ophthalmology	63 ophthalmologic residents	5-year various, surgical training related to ophthalmology	Surveys about the existing educational system	Post-intervention	To assess satisfaction with the current workplace and training	Likert scale questions, Multiple-choice questions, Free-response questions
Rocha et al. [30]	ATS Sch.	2025	Brazil	Coaching	Internal medicine	81 internal medicine residents	Lectures and virtual simulations about mechanical ventilation	Intervention for one group	Post-intervention	To assess learner satisfaction	Likert scale questions
Lefort et al. [31]	Pediatr Emerg Care.	2025	USA	Coaching	Disaster medicine	252 pediatric residents	10 modules about disaster medicine as a pediatric doctor	Intervention for one group	Post-intervention	To measure the module's acceptance	Likert scale questions

Settings and Interventions

The primary settings were coaching for residents or medical doctors (n = 12, 63%) [13,16,18,21-25,27,29-31], followed by basic medical education, such as lectures for medical students (n = 4, 21%) [15,19,20,28] and workplace-based learning (n = 3, 16%) [14,17,26]. Regarding the domain of educational interventions, emergency medicine was the most common (n = 6, 31%), followed by surgical disciplines (n = 4, 21%). In workplace learning settings, all three studies implemented educational interventions with electronic health records (EHRs) as the main theme. In most studies, the participants were medical doctors, whereas four focused on medical students. No distinct differences or trends were observed in the use, purpose, or interpretation of NPS across educational settings.

Application and Focus of NPS

The primary focus of NPS evaluations was educational intervention (n = 16, 84%) [14-21,23-31]. Other subjects of NPS evaluation were EHR systems in one study (5%) [14] and existing educational systems in three (16%) [13,22,29]. NPS was calculated collectively in all studies without categorizing participants by job roles. Educational intervention or the existing educational system was assessed after intervention without exception, whereas EHR was assessed in both pre- and post-intervention.

Associated Measures of NPS

Three studies [16,18,28] used NPS as a standalone measure, while four [14,19,21,25] combined it with additional evaluation metrics. Among the indicators used, only the modified simulation effectiveness tool (SET-M), explicitly designed to assess the effectiveness of simulation experiences, was suitable for evaluating educational interventions. Other indicators, such as emotional thriving/recovery/exhaustion, were unsuitable for evaluating the content of the educational intervention. Additionally, 13 studies [13,15,17,19-24,27,29-31] utilized questionnaires featuring rating scales or simple questions (both open and closed) to gather participants’ responses.

Comparison of NPS Usage Practices and Its Original Purpose

More than half of the selected studies utilized NPS to measure satisfaction. Only five studies [16-18,20,27] directly mentioned loyalty or recommendation, the original objectives of NPS, as reasons for its adoption. Thirteen studies (68%) adhered to the definition of NPS in terms of the question, and 10 studies (52%) clearly described calculation methods. Three studies altered how to answer NPS questions and calculate NPS, which led to failing to properly calculate NPS. The terms “satisfaction” and “loyalty” in relation to NPS were identified in 16 (84%) and five (26%) studies, respectively (see Table 3).

**Table 3:** Assessment related to Net Promoter Score (NPS)

Assessment item	Count, Percentage
The question/answer method is the original one.	13/19, 68%
The calculation method is the original one.	10/19, 52%
There are some descriptions of “satisfaction” related to NPS.	16/19, 84%
There are some descriptions of “loyal” related to NPS.	5/19, 26%

Discussion

Summary of Results

This scoping review identified 19 studies examining how NPS has been used as an evaluation tool in medical education. Most studies were conducted in the domain of coaching for medical doctors, such as residents, and only a few focused specifically on basic medical education for medical students. The content of educational interventions frequently focused on emergency medicine and surgery. Furthermore, the majority of studies (15 out of 19) were conducted in the United States, with the remaining four from Canada, Switzerland, France, and Brazil.

The Nature of NPS: Loyalty or Satisfaction

Across the included studies, NPS was most often used to measure general satisfaction with educational interventions, rather than its original purpose of assessing loyalty or willingness to recommend. When the purpose of NPS measurement focuses merely on satisfaction, which is a short-term emotion, the core objective of measuring loyalty may be overlooked, and the long-term impact and value of educational programs may be underestimated. Medical education is not a one-time experience but a continuous, cyclical process in which learners often become educators and pass on their knowledge to the next generation [32]. In this context, it is important to determine whether educational interventions are useful in the long term, given the current demand for improving the quality of medical education, and researchers should recognize that NPS is an indicator for measuring loyalty. Hence, we suggest that integrating both satisfaction-based and loyalty-focused interpretations, rather than focusing solely on satisfaction, may allow for a more meaningful use of NPS in evaluating educational programs that aim to foster lasting learning and professional growth.

Calculation Methods and Question Phrases of NPS

There was a lack of methodological consistency in calculation methods and question phrases. About half of the reviewed studies adhered to the original calculation method, with several failing to define how their score was derived clearly, and a few studies altered question phrases. This methodological fragmentation, as cautioned by a previous study, prevents meaningful comparison across research and weakens the metric's validity [33]. To resolve inconsistencies in NPS measurement, it is extremely important that standardized questions and calculation methods are disseminated and recognized by researchers. Improving consistency in how NPS is applied would enhance comparability across studies and allow for evaluation of intervention quality before and after changes.

Associated Measurements

While a few studies used NPS alongside demographic data, the majority combined it with additional feedback methods or general comments [4,5]. Although NPS has the limitation of not revealing the underlying reasons behind the score when used alone, most studies did not examine the reasons or explore what shaped participants’ responses. Understanding why some learners give low or high scores is essential for interpreting NPS meaningfully and using it to improve educational quality [7]. As suggested in earlier research on CME [9], examining the relationship between NPS and changes in learners’ attitudes, knowledge, or behavior may offer deeper insights into the effectiveness of educational interventions. In this review, NPS was measured with other metrics such as Likert scales, SET-M, and time-spent data; however, correlations with other metrics were not investigated. By examining free-text responses and the factors associated with NPS, it is possible to explore what types of educational interventions are needed. Similarly, investigating the correlation between NPS and learning outcomes, such as test scores, can help identify which indicators should be strengthened to improve NPS.

Study Setting

While this review included studies on medical students, unlike earlier work that focused only on CME for physicians [9], NPS was still not measured separately for medical students and medical professionals in any of the included studies. Additionally, the number of studies involving medical students was only four, suggesting that the use of NPS has not yet become sufficiently widespread in basic medical education compared to postgraduate education, thereby limiting the current depth of examination regarding the use and interpretation of NPS within the basic medical education field. In other fields, the impact of demographic characteristics on NPS has been investigated [34,35], but the effect of differences across stages of medical education (medical students, postgraduate, professional, etc.) on responses to NPS remains largely unexplored. Understanding how NPS responses vary across different learner groups could help identify which programs are most valued by specific audiences and support the development of more responsive and inclusive educational interventions.

Limitations

There are two limitations of this scoping review that should be noted. First, we included only peer-reviewed articles written in English. This may have excluded relevant studies published in other languages or gray literature, particularly those reflecting educational practices in non-Western contexts, which might lead to biased results. Moreover, as the previous research demonstrated, survey response styles are culturally dependent, and the presence of cultural artifacts implies that a simple comparison of NPS scores is vulnerable to bias [36]. Future reviews should therefore consider including non-English sources and local or community-based publications to provide a more global perspective and focus on cultural differences. Second, although the search strategy was systematic, we included only 19 studies, which may limit the generalizability of the findings. Expanding the search to include other healthcare professions and additional databases could explore the difference in NPS trends between different job types and yield more comprehensive results.

Implications for future research

The use of NPS in combination with objective measures such as test scores, skills assessments, or other quantitative indicators would enable researchers to investigate correlations between learners’ subjective evaluations and their actual performance outcomes. Such analyses could provide valuable insights into whether NPS reflects not only participants’ satisfaction or willingness to recommend but also the effectiveness of educational interventions in improving competence. Moreover, in studies that include both undergraduate medical students and postgraduate trainees, examining differences in NPS trends across groups may help explore how learners at different stages of training perceive and evaluate the same educational content.

In future research, it is desirable that NPS be applied using a consistent methodology [8], allowing for meaningful comparisons across educational interventions and quantitative systematic reviews. As more studies accumulate, it will become feasible to conduct scoping reviews that focus exclusively on research involving medical students. This would provide a more homogeneous evidence base for clarifying the role of NPS in undergraduate medical education. Moreover, broadening the scope to include other healthcare professionals as study participants may increase the number of eligible studies and facilitate the generation of more comprehensive or diverse findings, particularly regarding associated measures and their relationship with NPS.

## Conclusions

This scoping review revealed that the application of NPS in medical education frequently deviates from its original intent of measuring loyalty, with most studies using it instead to assess general satisfaction. In addition, inconsistencies were found in how NPS was phrased, calculated, and interpreted, and few studies provided insights into the reasons behind the scores or differences across learner subgroups. Exploring the factors underlying NPS and its correlations with quantitative indicators could provide valuable insights into trends in medical education and inform the future development of medical curricula. Furthermore, the clear dissemination of standardized NPS questions and scoring definitions will facilitate comparisons across educational programs, thereby broadening the applicability of NPS and enhancing its contribution to understanding trends in medical education.

## Appendices

Details of the Overall Screening Process and Reasons for Excluding Literature in Figure 1

In the identification stage, we identified duplicates based on DOI and title and removed 141 records. In the first screening, we removed 330 articles. Based on the title and abstract content, we determined whether studies were appropriate and excluded those that clearly did not meet the criteria, such as those focusing on non-medical industry themes or where subjects were primarily veterinarians or nurses. There were three reports we could not retrieve because they were not written in English.

In the second screening, we excluded 45 studies. The common reasons for exclusion were insufficient involvement of medical students or physicians as participants (19 studies) and a lack of focus on medical education or educational processes (14 studies). Additionally, several studies were excluded for evaluating clinical treatments or technologies rather than educational interventions. A typical exclusion case requiring discussion was a study investigating whether robotic total knee arthroplasty technology could be recommended as educational material for surgeons, and it was excluded because the educational intervention was not actually implemented.
